# Site specificity and attachment mode of *Symcallio* and *Calliobothrium* species (Cestoda: “Tetraphyllidea”) in smoothhound sharks of the genus *Mustelus* (Carcharhiniformes: Triakidae)

**DOI:** 10.7717/peerj.7264

**Published:** 2019-07-12

**Authors:** James P. Bernot, Janine N. Caira

**Affiliations:** 1 Institute for Biomedical Sciences, George Washington University, Washington, D.C., USA; 2 Department of Ecology & Evolutionary Biology, University of Connecticut, Storrs, CT, USA

**Keywords:** *Symcallio*, *Calliobothrium*, Tapeworm, Cestode, Attachment, Site specificity, Phylogeny, Laciniation, Laciniate, Strobila

## Abstract

Previous studies suggest that cestodes (i.e., tapeworms) of the sister genera *Symcallio* and *Calliobothrium* attach in different specific regions of the spiral intestine of their triakid shark hosts, with species of *Symcallio* attaching in the anterior region of the spiral intestine and species of *Calliobothrium* attaching with a broader distribution centered around the middle of the spiral intestine. In the present study, we tested the generality of this pattern of site specificity in two additional species pairs: *Symcallio peteri* and *Calliobothrium euzeti* in *Mustelus palumbes* and *S. leuckarti* and *C. wightmanorum* in *M. asterias*. Finding that these cestodes also exhibit the aforementioned pattern, we investigated a series of functional explanations that might account for this phylogenetically conserved pattern of site specificity. The mucosal surface of the spiral intestine of both shark species was characterized, as were the attachment mechanisms of all four cestode species. Although anatomical differences in mucosal surface were seen along the length of the spiral intestine in both shark species, these differences do not appear to correspond to the attachment mode of these cestodes. We find that while species of *Symcallio*, like most cestodes, attach using their scolex, species of *Calliobothrium* attach with their scolex and, to a much greater extent, also with their strobila. Furthermore, attachment of *Calliobothrium* species appears to be enhanced by laciniations (flap-like extensions on the posterior margins of the proglottids) that interdigitate with elements of the mucosal surface of the spiral intestine. The role of proglottid laciniations in attachment in species of *Calliobothrium* helps reconcile a number of morphological features that differ between these two closely related cestode genera.

## Introduction

Among the major groups of cestodes (i.e., tapeworms) that parasitize vertebrates, those found in elasmobranchs (i.e., sharks and rays) exhibit a disproportionately high degree of phylogenetic diversity, intensity of infection, and ubiquity, relative to the cestodes of other vertebrate groups. In total, cestodes of elasmobranchs represent nine of the 19 cestode orders currently recognized and parasitize members of all 11 orders of elasmobranchs (Caira & Jensen, 2014, 2017). They are typically moderate in size, with most species ranging in total length (TL) from 500 µm to 60 cm (Caira & Jensen, 2014). Adults of the majority of elasmobranch cestodes live in the spiral intestine—a specialized organ, unique to elasmobranchs and a few bony fish, that combines the functions of the small and large intestines in a single structure. Most elasmobranch cestodes are highly host-specific (Caira & Jensen, 2014). As is the case for the majority of cestodes, they attach to the surface of the mucosa of their host with a specialized attachment organ at the anterior end of their body referred to as a scolex.

**Figure 1:**
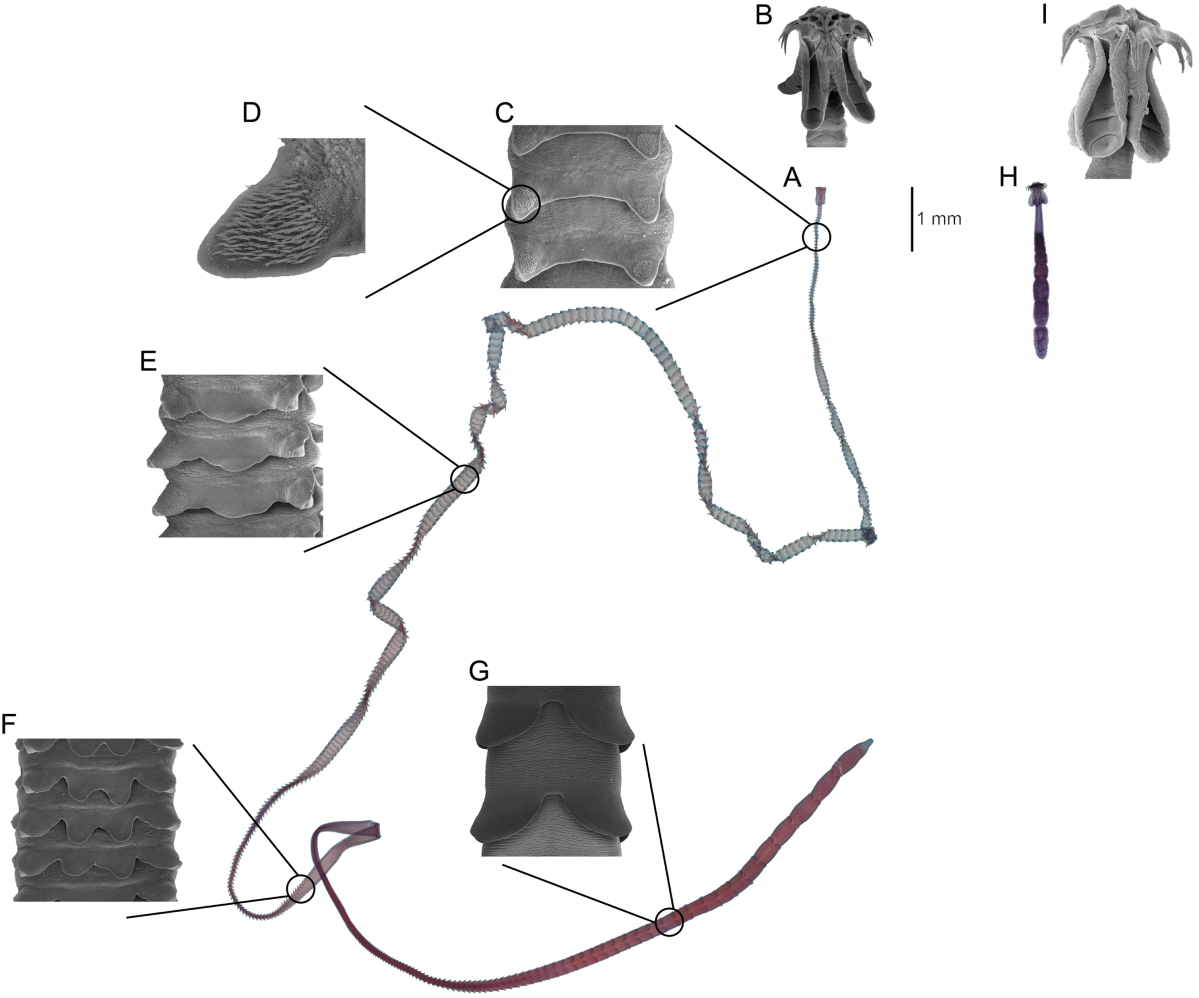
Comparison of major differences between *Calliobothrium* and *Symcallio*. *Calliobothrium* (A) Light micrograph of whole worm. (B) Scanning electron micrograph (SEM) of scolex. (C) SEM of immature proglottids with two laciniations. (D) SEM detailing spinithrix patch on proglottid laciniation in C. (E) SEM of immature proglottid with three laciniations. (F) SEM of immature proglottids with four laciniations. (G) SEM of mature proglottids with two laciniations. *Symcallio* (H) Light micrograph of whole worm, shown at same scale as A. (I) SEM of scolex, shown at same scale as B.

This paper focuses on the phylogenetically conserved pattern of site specificity observed across multiple, highly host-specific species in the cestode genera *Symcallio* Bernot, Caira, and Pickering 2015 and *Calliobothrium* van Beneden, 1850 that parasitize the spiral intestine of sharks of the genus *Mustelus* Linck, 1790 (Nasin, Caira & Euzet, 1997; Ivanov & Brooks, 2002; Kurashima et al., 2014; Bernot, Caira & Pickering, 2015, 2016). Typically, each species of *Mustelus* is parasitized by both a unique species of *Symcallio* and a unique species of *Calliobothrium*. Beyond a scolex that consists of four muscular bothridia each armed with two pairs of hooks (Figs. 1B and 1I), the genera are morphologically quite divergent. Whereas species of *Calliobothrium* are large, slender worms (Fig. 1A), with a small scolex (Fig. 1B), species of *Symcallio* are small worms (Fig. 1H), with a relatively robust scolex (Fig. 1I). While the numerous proglottids comprising the strobila (i.e., the chain of proglottids that make up the body posterior to the scolex) of *Calliobothrium* each bear a series of posterior projections (Figs. 1C–1G) referred to as laciniations, those of *Symcallio* do not. Members of the two genera also differ in attachment site. While species of *Symcallio* attach in the anterior region of the spiral intestine, those of *Calliobothrium* have a broader distribution centered around the middle of the spiral intestine. This pattern has been documented for the members of both cestode genera in *Mustelus mustelus* (L., 1758) by Euzet (1959), *M. canis* (Mitchell, 1815) by Cislo & Caira (1993), and *M. schmitti* Springer, 1939 by Alarcos, Ivanov & Sardella (2006).

Nonetheless, the factors that might account for the conserved site specificity of each genus have yet to be identified. As suggested by those working in other cestode/elasmobranch systems (Williams, 1966, 1968; Williams, McVicar & Ralph, 1970; Carvajal & Dailey, 1975; Borucinska & Caira, 1993), Cislo & Caira (1993) hypothesized that the species of *Symcallio* and *Calliobothrium* parasitizing *M. canis* may be able to attach solely to mucosal surfaces with configurations that complement the morphologies of their respective scoleces. However, these authors noted that characterization of the mucosal surface of the spiral intestine of *M. canis* was required to address this hypothesis.

This paper aims to investigate the hypothesis advanced by Cislo & Caira (1993) to explain the pattern of site specificity seen in members of these two cestode genera. It has three main goals. First, to test the generality of the pattern of site specificity in two additional pairs of *Symcallio* and *Calliobothrium* species parasitizing two additional species of *Mustelus*: *Symcallio peteri* Bernot, Pickering, and Caira, 2015 and *Calliobothrium euzeti* Bernot, Pickering, and Caira, 2015 in *M. palumbes* Smith, 1957 and *S. leuckarti* (van Beneden, 1850) Bernot, Caira, and Pickering, 2015 and *C. wightmanorum* Bernot and Caira, 2016 in *M. asterias* Cloquet, 1819. Second, to characterize the configuration of the mucosal surface of the spiral intestines of both species of *Mustelus*. Third, to investigate the mode of attachment to those surfaces by species of *Symcallio* and *Calliobothrium* found parasitizing each species of *Mustelus*.

### Materials and Methods

All sharks examined were assigned a unique identification code and basic measurements and a series of digital photographs were taken. These data are available using the host collection code and collection number (e.g., UK-3) in the Global Cestode Database at http://elasmobranchs.tapewormdb.uconn.edu (Caira, Jensen & Barbeau, 2018). In addition, a sample of liver tissue was removed from each shark specimen and preserved in 95% ethanol for future molecular verification of host identifications (see Naylor et al., 2012). In total, five specimens of *M. palumbes* were collected by bottom trawl in the Indian Ocean off South Africa (between 33°3.36′S to 36°25.97′S and 21°25.73′E to 26°38.96′E) in April and May of 2010 as by-catch from a hake survey conducted by the Marine and Coastal Management, Department of Agriculture, Forestry and Fisheries, South Africa on the FRS *Africana*. Specimens examined consisted of three males (AF-41, AF-120, AF-122) 68–83 cm TL and two females (AF-168, AF-169) 67–69 cm TL. In addition, 11 specimens of *M. asterias* were collected by long-line in the North Sea off Lowestoft, Great Britain in August of 2013 by the FV *Maximus* working in conjunction with the Centre for Environment, Fisheries and Aquaculture Science (Cefas) of the United Kingdom. Specimens examined consisted of nine males (UK-3, UK-4, UK-9, UK-14, UK-25, UK-27, UK-28, UK-29, and UK-32) 80–99 cm in TL and two females (UK-36 and UK-52) 77.5–80 cm TL. All animals used in this study were handled in accordance with the ethics protocols of the University of Connecticut’s Institutional Animal Care and Use Committee (IACUC numbers A08-044 and A11-030). Shark taxonomy follows Compagno, Dando & Fowler (2005).

The spiral intestine was removed from each shark and opened following the standard method used in similar studies (Cislo & Caira, 1993; Pickering & Caira, 2008, 2014; Twohig, Caira & Fyler, 2008; Bernot, Caira & Pickering, 2015, 2016). A midventral incision was made along the right side of the ventral vein cutting from anterior to posterior so as to also cut the internal spiraling mucosal tissue into the same number of chambers, visible as flaps of the internally spiraling tissue, with the same relative size across specimens. Spiral intestines were fixed in seawater-buffered formalin (9:1, seawater:full-strength formalin) for approximately 1 week and then transferred to 70% ethanol for storage. The surface of each chamber of the spiral intestine was examined using a dissecting microscope, all cestodes were systematically removed, and the chamber of attachment recorded. Only those cestodes found attached to the mucosa were included in this study.

Histological sections of worms in situ were prepared for two specimens each of *C. euzeti* Bernot, Caira, and Pickering, 2015 and *S. peteri* Bernot, Caira, and Pickering, 2015 from *M. palumbes*, and two specimens of *C. wightmanorum* Bernot and Caira, 2016 from *M. asterias* as follows. The scolex of each worm and an approximately one cm^2^ piece of the mucosal tissue surrounding the scolex and its associated strobila was cut from the spiral intestine. These samples were dehydrated in a graded ethanol series, cleared in xylene, and embedded in a paraffin-polymer blend (TissuePrep; Fisher Scientific, Fair Lawn, NJ, USA). Cross sections, made parallel to the mucosal surface, were cut at seven μm intervals using an Olympus Cut 4060 microtome (Olympus Corporation, Melville, NY, USA). Sections were attached to glass slides with warm 10% aqueous sodium silicate, hydrated in a graded ethanol series, stained with Delafield’s hematoxylin, counterstained with eosin, dehydrated in a graded ethanol series, cleared in xylene, and mounted in Canada balsam on glass slides under coverslips. Histological sections were examined, measured, and photographed with a Zeiss Axioskop 2 Plus microscope (Zeiss, Thornwood, NY, USA) using a SPOT Diagnostic Instrument Digital Camera System and SPOT software (version 4.6) (SPOT Imaging Solutions, Sterling Heights, MI, USA). To confirm cestode identifications, a subset of the worms removed was prepared as whole mounts in Canada balsam using standard protocols (see Pickering & Caira, 2012) and examined with light microscopy.

Scanning electron microscopy (SEM) was also used to characterize the mucosal surface of the spiral intestine and to investigate the mode of attachment of both *Calliobothrium* species. To evaluate the structure of the mucosal surface of the intestine, three pieces of mucosal tissue measuring approximately one cm^2^ were cut from chambers 1, 4, and 8 (anterior, middle, and posterior) of the spiral intestine of one specimen of *M. palumbes*. To examine the attachment mechanism of *Calliobothrium* species, two in situ specimens of *C. euzeti* and one in situ specimen of *C. wightmanorum*, along with approximately one cm^2^ of the surrounding mucosal tissue, were cut from the spiral intestines of their respective hosts and prepared for SEM. Specimens were hydrated in a graded ethanol series, placed in 1% osmium tetroxide overnight, dehydrated in a graded ethanol series, placed in hexamethyldisilazane (Ted Pella Inc., Redding, CA, USA), and allowed to air dry in a fume hood. They were subsequently mounted on aluminum stubs using double-sided adhesive PELCO carbon tabs (Ted Pella Inc., Redding, CA, USA), sputter coated with 30–60 nm of gold/palladium, and examined with an FEI Nova NanoSEM 450. The stubs were retained in the personal collection of the second author.

Histological sections and SEM specimen vouchers were deposited in the Lawrence R. Penner Parasitology Collection (LRP), Department of Ecology and Evolutionary Biology, University of Connecticut, Storrs, Connecticut, USA. Specimens deposited are as follows: serial histological sections of one in situ specimen of *S. peteri* (19 slides, LRP Nos. 9964–9982); serial histological sections of one in situ specimen of *C. euzeti* (20 slides, LRP Nos. 9983–10002); serial histological sections of one in situ specimen of *C. wightmanorum* (11 slides, LRP Nos. 10004–10014); vouchers of three paratypes of *C. wightmanorum* examined with SEM (LRP Nos. 8885–8887).

## Results

### Anatomy of the spiral intestine

**Figure 2:**
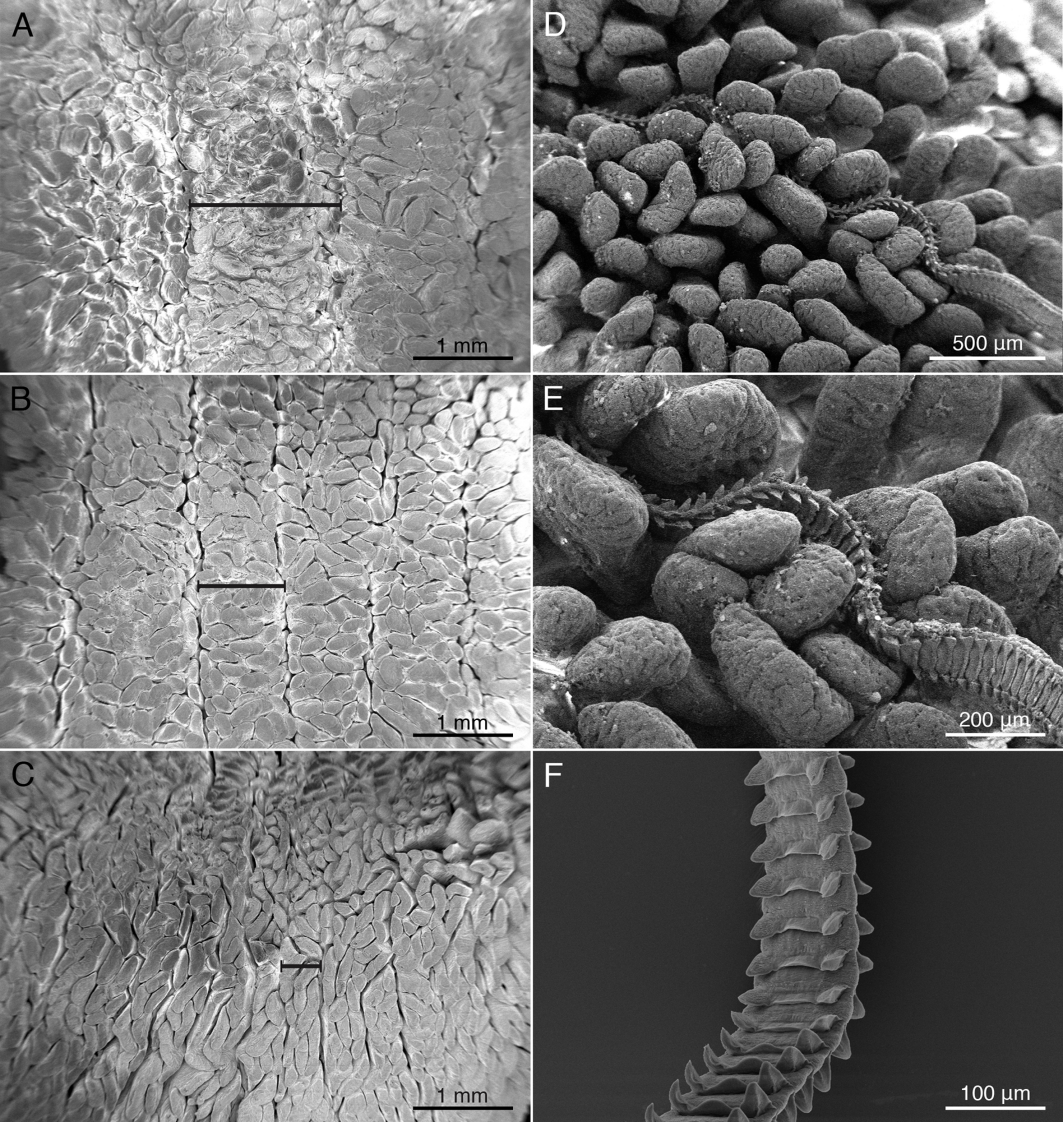
Scanning electron micrographs (SEM) of mucosal surface of spiral intestine and cestodes. Major mucosal elements visible as large ridges (black dimension lines in (A–C); minor mucosal elements visible as small villi. *Mustelus palumbes* mucosal surface of spiral intestine (A) Chamber 1. (B) Chamber 4. (C) Chamber 8. (D) SEM of mucosal surface of chamber 3 of spiral intestine of *M. palumbes* with *Calliobothrium euzeti* attached. (E) Detail of D. (F) SEM of anterior region of strobila of *Calliobothrium wightmanorum*.

The spiral intestine of *M. palumbes* was found to consist of nine auger-like turns of the internally spiraling mucosa. When opened as described above, these turns correspond to nine chambers. Two basic types of mucosal surface elements are present. The mucosal surface throughout the entire length of the spiral intestine is covered with small, elongate, villus-like projections (Figs. 2A–2C). The bases of neighboring villi were found to be fused together near the proximal surface of the mucosa. In addition, the mucosal surface has large longitudinal folds, visible as ridges, for much of the anterior half of the spiral intestine. These ridges are widest in the first chamber (Fig. 2A) and become progressively narrower in the subsequent chambers, such that their width in the fourth chamber (Fig. 2B) is only about half their width in the first chamber; these ridges are almost undetectable in the posterior three chambers (Fig. 2C).

The spiral intestine of *M. asterias* was found to consist of 10 auger-like turns of the mucosa that correspond to 10 chambers. The mucosa exhibits two basic types of surface elements. Small, elongate, villus-like projections that are fused together at their base are present throughout the mucosal surface of the entire spiral intestine. Throughout much of the anterior half of the spiral intestine the mucosal surface is thrown into longitudinal folds, visible as large ridges. The ridges are widest in the first chamber and become progressively narrower in subsequent chambers; they are virtually absent in the three posterior-most chambers.

### Distribution of cestodes within the spiral intestine of *M. palumbes*

**Figure 3:**
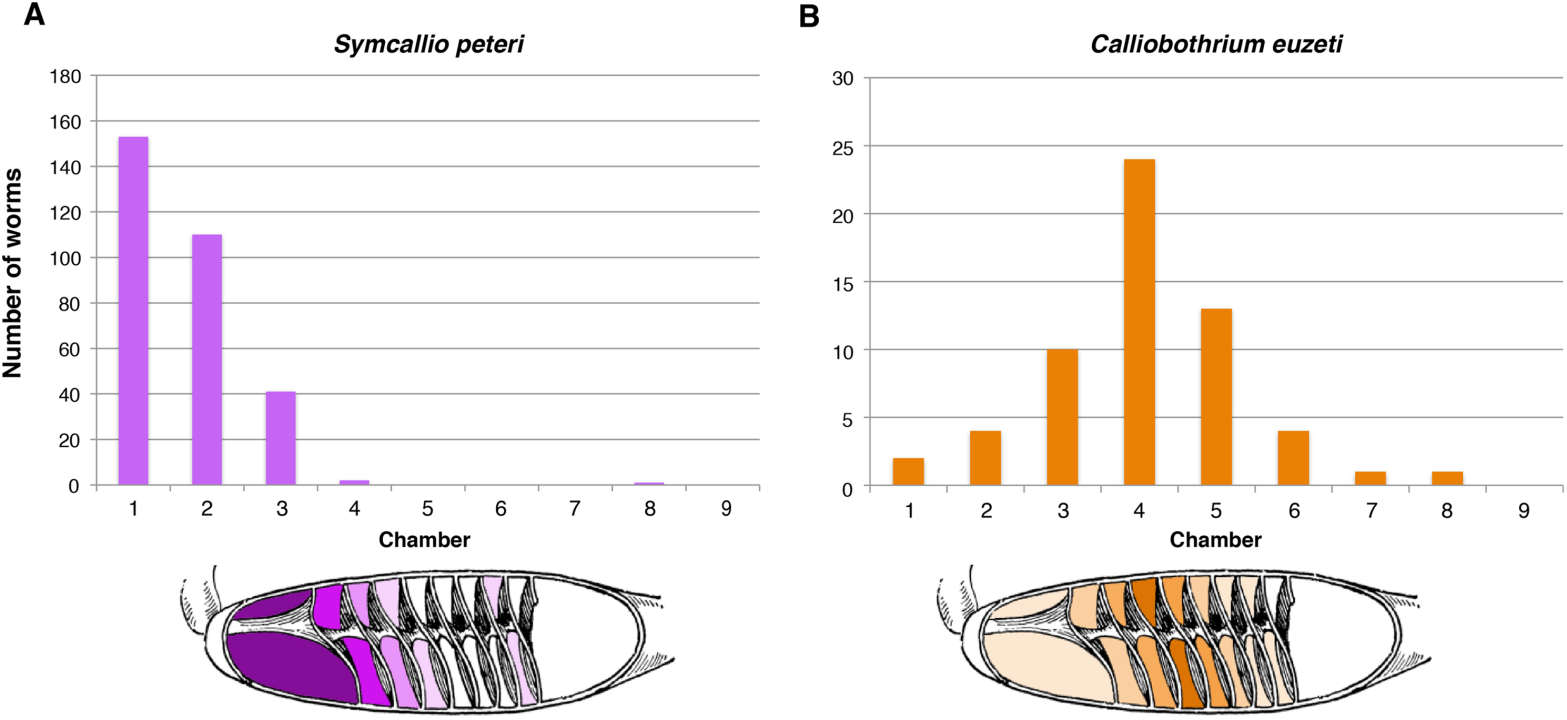
Total number of attached specimens recovered from each chamber of the spiral intestine in five individuals of *Mustelus palumbes*. (A) *Symcallio peteri*. (B) *Calliobothrium euzeti*.

The five specimens of *M. palumbes* examined were found to host a total of 308 attached specimens of *S. peteri* and 59 attached specimens of *C. euzeti*. The chambers occupied by these worms are summarized in Fig. 3. Nearly all specimens of *S. peteri* were found attached in the anterior three chambers (i.e., *n* = 305, 99%); two specimens were found in chamber 4 and a single specimen was found in chamber 8 (Data S1). Approximately half (*n* = 153, 49.8%) of the specimens of *S. peteri* were found in the first chamber. Specimens of *C. euzeti* were more broadly distributed. All 59 specimens were attached in chambers 1–8; the majority of specimens (i.e., 47, 80%) were attached in chambers 3–5.

### Distribution of cestodes within the spiral intestine of *M. asterias*

**Figure 4:**
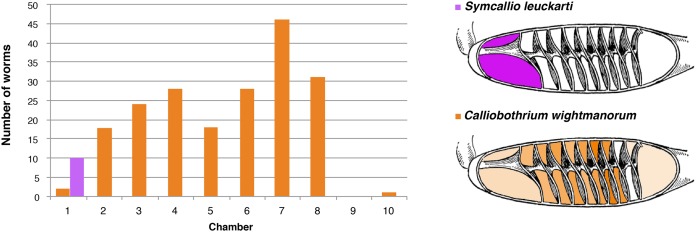
Total number of attached specimens of *Symcallio leuckarti* and *Calliobothrium wightmanorum* recovered from each chamber of the spiral intestine in 11 individuals of *Mustelus asterias*.

The 11 specimens of *M. asterias* examined were found to host a total of 10 attached specimens of *S. leuckarti* and 196 attached specimens of *C. wightmanorum*. Figure 4 summarizes the chambers occupied by the worms of both species. All 10 specimens of *S. leuckarti* were attached in the first chamber of the spiral intestine. *C. wightmanorum* was found broadly distributed throughout chambers 1–10. The number of specimens per chamber ranged from 46 (23%) in chamber 7 to 1 (0.5%) in chamber 10 (Data S2).

### Mode of attachment

#### Symcallio peteri

**Figure 5:**
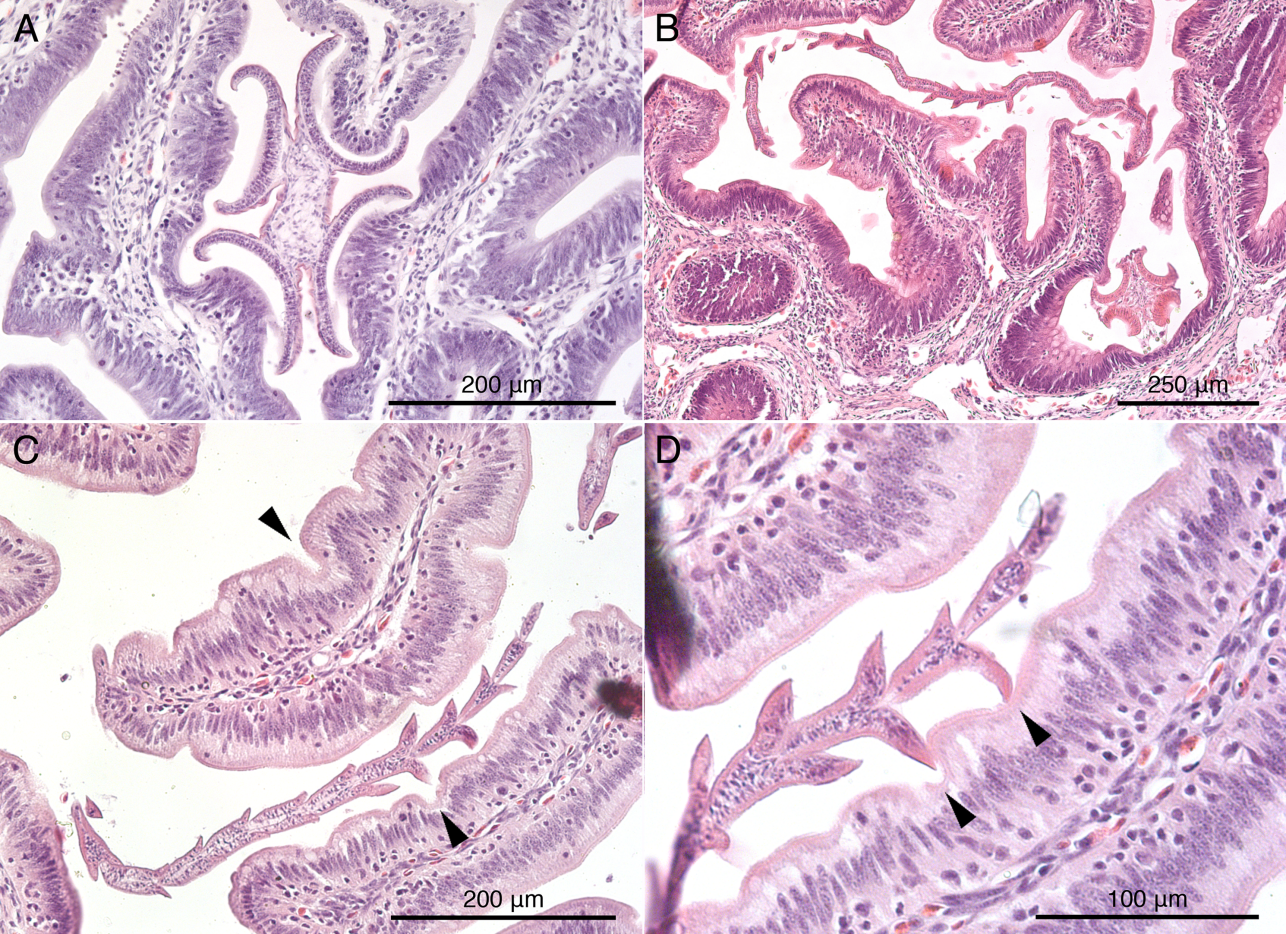
Histological sections of mucosal surface of *Mustelus palumbes* with cestodes attached. (A) *Symcallio peteri* scolex. (B) *Calliobothrium euzeti* scolex and strobila. (C) *C. euzeti* strobila. (D) *C. euzeti* proglottid laciniations. Arrowheads in (C) and (D) indicate dimples in surface of villi.

Histological sections of worms in situ indicate that *S. peteri* inserts its scolex between the villi of the mucosal surface of *M. palumbes*, grasping portions of adjacent villi with each of its four muscular bothridia (Fig. 5A). Its hooks are embedded into the mucosa at their points of contact with the surface. Its short strobila (Fig. 1H) extends freely into the lumen of the spiral intestine.

#### Calliobothrium euzeti

Histological sections of *C. euzeti* in situ demonstrate that this species inserts its scolex between the villi of the mucosal surface of *M. palumbes*. It grasps portions of adjacent villi with its four muscular bothridia (Fig. 5B) and its hooks are embedded at their points of contact with the mucosal surface. In addition, much of the anterior half of the strobila was observed, both under a dissecting microscope and with SEM, to be interlaced among the villi of the mucosa (Figs. 2D and 2E). Histological sections indicate the laciniations of the proglottids interdigitate with small dimples on the surface of the villi (Figs. 5C and 5D); these dimples were also visible with SEM (Fig. 2E). The remainder of the body, consisting of more mature proglottids with broader and flatter laciniations (Fig. 1G), extends freely into the lumen of the spiral intestine.

#### Symcallio leuckarti

Owing to the limited number of specimens of *S. leuckarti* collected, these specimens were used in the redescription of this species by Bernot, Caira & Pickering (2016) rather than for in situ histological sections here. However, examination of specimens of *S. leuckarti* in situ under a dissecting microscope at the time of their removal revealed that this species also inserts its scolex among the villi of the spiral intestine of *M. asterias*. Specimens were seen grasping adjacent villi with each of the four muscular bothridia on their relatively large scolex while the strobila extended freely into the lumen of the spiral intestine.

#### Calliobothrium wightmanorum

Histological sections of specimens in situ (images not shown) indicate that *C. wightmanorum* attaches with its scolex inserted between the villi of the mucosal surface of *M. asterias*, grasping portions of adjacent villi with its four muscular bothridia and embedding its hooks in the mucosa at their points of contact with that surface. Nearly one-half of the anterior portion of the strobila was found interlaced among the villi of the mucosal surface. Histological sections revealed dimple-like structures on the villi in *M. asterias* with which the laciniations of this species (Fig. 2F) appear to interdigitate.

## Discussion

As has been found in many other groups of elasmobranch tapeworms (Euzet, 1959; Williams, 1966, 1968; Williams, McVicar & Ralph, 1970; Carvajal & Dailey, 1975; Borucinska & Caira, 1993; Caira & Runkle, 1993; McKenzie & Caira, 1998; Twohig, Caira & Fyler, 2008), species of *Symcallio* and *Calliobothrium* were found to attach to the mucosal surface of their hosts using their scolex. McKenzie & Caira (1998) recognized two main modes of attachment to the mucosa in elasmobranch cestodes. “Jamming,” in which the bothridia of the cestode are intercalated between elements of the mucosal surface (e.g., villi) and exert pressure on the sides of these mucosal structures, leaving the distal tips of the mucosal elements essentially free; and “grabbing,” in which each bothridium attaches to the tip of one or more villus-like elements of the mucosal surface. Species of *Calliobothrium* and *Symcallio* both appear to exhibit the “jamming” mode of attachment wherein their scolex is wedged between the villi of the mucosal surface and their muscular bothridia are used to grasp the sides of these small mucosal elements (Figs. 5A and 5B).

Somewhat unexpectedly, both species of *Calliobothrium* were also found to use the immature proglottids composing the anterior portion of the strobila to attach to the surface of the mucosa of their respective hosts. Nearly one-third to one-half of the strobila of each worm was observed to be interwoven among the villi of the mucosal surface. Furthermore, the laciniations of the posterior margins of the immature proglottids were found to act as additional anchoring points to help these worms attach even more securely to the mucosal surface. These laciniations fit into small dimples on the surfaces of the villi in the spiral intestine of both species of smoothhound sharks examined here. This is a highly effective strategy given that the spacing of adjacent dimples on the villi of *M. palumbes* appears to correspond closely with the length of the immature proglottids of *C. euzeti*, and thus also the spacing of laciniations on consecutive immature proglottids (Fig. 5D). This leads us to suspect that the laciniations seen in members of other elasmobranch-parasitizing cestode genera, such as *Crossobothrium*, and possibly *Anthobothrium*, also assist members of these genera with attachment to the mucosal surface of their hosts.

The role laciniate proglottids are now known to play in aiding with attachment of *Calliobothrium* species sheds light on other previously puzzling morphological features. First, it helps to reconcile the diminutive size of the scolex of *Calliobothrium* species relative to that of their strobila (Figs. 1A, 1B vs 1H, 1I). Given this discrepancy, it seems unlikely that such a tiny scolex could serve to effectively anchor the large bodies of members of this genus to the mucosal surface in the hostile, active environment of the spiral intestine. What we have demonstrated here is that the scolex alone does not do so. Attachment is enhanced by the anterior half of the strobila and the laciniations of immature proglottids. This may also help explain the conspicuously large number of immature proglottids relative to mature proglottids seen in *Calliobothrium* species, which typically possess nearly 300 immature proglottids and less than 10 mature proglottids. Given the laciniations of immature proglottids are more well developed for attachment than those of mature proglottids (Figs. 1C–1F vs 1G), it seems likely that these worms maintain large numbers of immature proglottids in order to remain more firmly attached to their hosts.

The role of laciniations in attachment also helps to explain the presence of spine-like projections of the tegument, referred to as gladiate spinitriches, observed on the surface of the laciniations of multiple species of *Calliobothrium* (e.g., *C. australis* Ivanov and Brooks, 2002 fig. 16; *C. euzeti* Bernot et al., 2015 fig. 8D, E; *C. cisloi* Bernot et al., 2016 fig. 2E, F; *C. wightmanorum* Bernot et al., 2016 fig. 4D, E). These projections, which are normally found on the scolex of cestodes, have been implicated in assisting with attachment (Chervy, 2009). Now that laciniations are known to aid in attachment, it seems reasonable to believe the gladiate spinitriches on laciniations (Figs. 1C–1F) of *Calliobothrium* species assist with this process.

In contrast, despite bearing a body that is one to two orders of magnitude shorter than those of most *Calliobothrium* species (Figs. 1H vs 1A), *Symcallio* species exhibit a scolex that is approximately twice the size of that of *Calliobothrium* species (Figs. 1I vs 1B). Given that *Symcallio* species lack proglottid laciniations and the entire strobila extends freely into the lumen of the intestine, it seems likely that the relatively large scolex of *Symcallio* species bears the entire weight of the body.

The sites occupied by the species of *Symcallio* and *Calliobothrium* in both *M. palumbes* and in *M. asterias* were found to be consistent with the pattern exhibited by their congeners in other species of *Mustelus* (see Euzet, 1959; Cislo & Caira, 1993; Alarcos, Ivanov & Sardella, 2006). All seven species of *Symcallio* for which attachment site data are now available attach primarily in the anterior three chambers of the spiral intestine of their respective hosts. The five species of *Calliobothrium* for which attachment site data now exist attach more broadly across the length of the spiral intestine, with a distribution that is centered around the middle chambers of the organ. This provides further support for the notion that there is a phylogenetic component to attachment site specificity in what Bernot, Caira & Pickering (2016) determined are sister genera. If so, it seems likely that the remaining seven described species of *Symcallio* will also be found to attach primarily in chambers 1–3 whereas the other four described species of *Calliobothrium* will have a broader distribution with most individuals found in the central chambers of the spiral intestine. Given that 16 species of *Mustelus* have never been examined for species of *Symcallio* and that 22 species of *Mustelus* have not been examined in detail for species of *Calliobothrium*, we predict that the 16 new species of *Symcallio* and the 22 new species of *Calliobothrium* we anticipate will be discovered in these hosts will exhibit a similar pattern of site specificity.

Our work leads us to reject the hypothesis advanced by Cislo & Caira (1993) that a correspondence between scolex morphology and mucosal surface topology at the site of attachment might explain the differences in site of attachment seen between *Symcallio* and *Calliobothrium* species. Mucosal surface differences were seen along the length of the spiral intestines of both *M. palumbes* and *M. asterias* in that the villus-bearing mucosal surface of the anterior-most chambers was observed to be thrown into large ridge-like folds that became less and less pronounced in the more posterior chambers. However, these ridges exist at a scale that does not appear to be relevant to attachment for either cestode genus. Not only were the species in both genera found to engage with the small villi, rather than the large ridges, at their sites of attachment, but also these small villi were found throughout the surfaces of the mucosa in all chambers of the spiral intestines of both shark species. As a consequence, mucosal surface topology does not explain the fact that *Symcallio* species attach predominantly in the three anterior-most chambers of the spiral intestines of their respective hosts. Although attachment to the villi accounts for the fact that *Calliobothrium* species are found throughout the length of the spiral intestine, it does not explain why the majority of specimens of members of this genus are concentrated in the middle chambers of the spiral intestine.

Other factors that might account for this phylogenetically conserved site specificity are interesting to consider. Cestodes lack all elements of a digestive system; they absorb their nutrients from the digestive system of their hosts. It is possible that members of the two genera differ in their nutritional needs and that the resources provided by species of *Mustelus* differ along the length of their spiral intestines. Unfortunately, little is known about the nutrient requirements of cestodes overall. It appears they are unable to synthesize fatty acids and cholesterol de novo and also lack the ability to synthesize some amino acids, instead depending on their hosts for these nutrients (Tsai et al., 2013). While studies on the physiology of the spiral intestine of sharks are generally lacking, studies on metabolism and absorption of nutrients along the digestive tract of bony fish show that different nutrients (e.g., sugars, amino acids, and fatty acids) are metabolized in different regions of the intestine (Bakke, Glover & Krogdahl, 2010). It would be interesting to explore absorption and nutrient concentrations along the length of the spiral intestine of species of *Mustelus*. The detection of consistent regional differences across members of the genus would lend support to this hypothesis. However, it is equally important to examine whether the nutritional needs of species of *Symcallio* and species of *Calliobothrium* are similar within a genus, but different between genera.

It is also possible that site specificity is more relaxed in *Calliobothrium* species because members of this genus are essentially one to two orders of magnitude longer than species of *Symcallio* (Figs. 1A and 1H). As a result, much of the strobila of members of the former genus extends into the lumen across multiple chambers of the spiral intestine, making the actual site of attachment of the scolex less important. In contrast, the small bodies of *Symcallio* species rarely extend more than a few millimeters beyond the attachment point of their scoleces. This difference in size may be important not only in terms of nutrient availability, but also in the context of reproductive efficiency. In order for individuals of *Symcallio* to sexually reproduce, their scoleces must be in much closer proximity relative to the much longer individuals of *Calliobothrium*. Furthermore, whereas species of *Symcallio* are apolytic, retaining gravid proglottids on the strobila, species of *Calliobothrium* are generally euapolytic, dropping mature proglottids from the strobila. These free proglottids of *Calliobothrium* species are capable of independent movement and thus have the ability to find and mate with other free proglottids independent of the strobila.

It is also possible that the differential site specificity exhibited by *Symcallio* and *Calliobothrium* species has an ecological basis. Given that species from each genus frequently co-occur in the same host individual, selective pressure from competition to reduce niche overlap could explain their different attachment sites. However, Cislo & Caira (1993) did not find evidence of competitive interaction between *S. violae* and *C. cisloi* (identified therein as *C. lintoni* and *C. verticillatum*, respectively) in *M. canis*, and thus rejected competition as a factor accounting for the different attachment sites in these species. Nonetheless, it is possible that the presently observed site segregation of *Symcallio* and *Calliobothrium* is the evolutionary end-product of past competition, a process also referred to as “the ghost of competition past” (Connell, 1980; Price, 1987). Alternatively, *Symcallio* and *Calliobothrium* could have evolved independently in different host species and adapted to different environmental conditions, such that when they later colonized the same host, they occupied the sites to which they had already adapted. To further elucidate ecological factors that may contribute to the site specificity of these genera, a more detailed understanding of the phylogeny of *Symcallio* and *Calliobothrium* is needed and, ideally, also experimental studies (see Connell, 1980; Price, 1987).

## Conclusions

This study has led us to consider the interface between cestodes and their hosts at a different scale. It was the small villi of the mucosa, present in the same form throughout the length of the spiral intestine, rather than the major ridges that varied in size along the length of the intestine, that were found to play a major role in the attachment of both genera of cestodes examined here. Perhaps the driver of site specificity in this system also exists at an even finer scale, such as the nutritional resources available throughout the length of the labile environment of the spiral intestine. The consistency of the pattern across multiple species in both cestode genera suggests that the factors responsible have their origin somewhere in the evolutionary history of the two genera, rather than in more recent times.

## Supplemental Information

10.7717/peerj.7264/supp-1 Supplemental Information 1 *Mustelus palumbes* cestode site data. Number of attached cestodes recovered from each chamber of spiral intestine of five individuals of *M. palumbes*

10.7717/peerj.7264/supp-2 Supplemental Information 2 *Mustelus asterias* cestode site data. Number of attached cestodes recovered from each chamber of the spiral intestine of 11 individuals of *M. asterias*

## Additional Information and Declarations

### Data Availability

The following information was supplied regarding data availability: The raw counts of specimens removed from each chamber of the spiral intestine of each specimen of *Mustelus palumbes* and *Mustelus asterias* are available in the Supplemental Files. Histological sections and SEM specimen vouchers were deposited in the Lawrence R. Penner Parasitology Collection (LRP), Department of Ecology and Evolutionary Biology, University of Connecticut, Storrs, Connecticut, USA. Specimens deposited are as follows: serial histological sections of one in situ specimen of *Symcallio peteri* (19 slides, LRP Nos. 9964–9982); serial histological sections of one in situ specimen of *Calliobothrium euzeti* (20 slides, LRP Nos. 9983–10002); serial histological sections of one in situ specimen of *Calliobothrium wightmanorum* (11 slides, LRP Nos. 10004–10014); vouchers of three paratypes of *C. wightmanorum* examined with SEM (LRP Nos. 8885–8887). SEM stubs were retained in the personal collection of the second author and are preserved and stored at the University of Connecticut Electron Microscopy Lab under vacuum where they are available for interested parties to examine.
